# Supplementary material for: CD44v6 High Membranous Expression Is a Predictive Marker of Therapy Response in Gastric Cancer Patients
Source: Biomedicines. 2021 Sep 18;9(9):1249. doi: 10.3390/biomedicines9091249 (PMC8465138; doi:10.3390/biomedicines9091249)
Supplement: Supplementary file 1 [file biomedicines-09-01249-s001.zip › biomedicines-1366449-supplementary.pdf]

Supplementary Materials

CD44v6 high membranous expression is a predictive marker of therapy response in gastric cancer patients

Gabriela M Almeida [1,2,3,†], Carla Pereira [1,2,4,†], Ji-Hyeon Park [5], Carolina Lemos [1,6,7], Sofia Campelos [1,8], Irene Gullo [1,3,9], Diana Martins [1,2,10], Gilza Gonçalves [2], Dina Leitão [3,9], João Luís Neto [11], Ana André [1,2], Clara Borges [12], Daniela Almeida [12], Hyuk-Joon Lee [5,13,14], Seong-Ho Kong [5,14], Woo Ho Kim [15], Fátima Carneiro [1,2,3,9], Raquel Almeida [1,2,3,16], Han-Kwang Yang [5,13,14] and Carla Oliveira [1,2,3,*]

[1] i3S–Instituto de Investigação e Inovação em Saúde, Universidade do Porto, 4200-135 Porto, Portugal; galmeida@ipatimup.pt (G.M.A.); clrc.pereira8@gmail.com (C.P.); clclemos@ibmc.up.pt (C.L.); Sofia.campelos@gmail.com (S.C.); irene.gullo12@gmail.com (I.G.); dianam@ipatimup.pt (D.M.); aandre@ipatimup.pt (A.A.); fcarneiro@ipatimup.pt (F.C.); ralmeida@ipatimup.pt (R.A.)

[2] Ipatimup–Institute of Molecular Pathology and Immunology of the University of Porto, 4200-135 Porto, Portugal; gilzasofia@gmail.com (G.G.)

[3] Faculty of Medicine, University of Porto, 4200-319 Porto, Portugal; dinaraquel@med.up.pt (D.L.)

[4] Doctoral Programme on Biomedicine, Faculty of Medicine, University of Porto, Porto, Portugal

[5] Department of Surgery, Seoul National University Hospital, 03080 Seoul, Korea; pjhaaa1220@gmail.com (J.-H.P.); appe98@snu.ac.kr (H.-J.L.); wisehearted@gmail.com (S.-H.K.); hkyang@snu.ac.kr (H.-K.Y.)

[6] UnIGENe, IBMC–Institute for Molecular and Cell Biology, 4200-135 Porto, Portugal

[7] ICBAS–Instituto Ciências Biomédicas Abel Salazar, Universidade do Porto, 4050-313 Porto, Portugal

[8] Department of Pathology, Ipatimup Diagnostics, Institute of Molecular Pathology and Immunology, University of Porto, 4200-135 Porto, Portugal

[9] Department of Pathology, Centro Hospitalar Universitário de São João, 4200-319 Porto, Portugal

[10] Department of Biomedical Laboratory Sciences, ESTeSC–Coimbra Health School, Polytechnic Institute of Coimbra, 3046-854 Coimbra, Portugal

[11] Instituto de Medicina Molecular João Lobo Antunes, Faculdade de Medicina, Universidade de Lisboa, 1649-028 Lisbon, Portugal; joaoneto@medicina.ulisboa.pt

[12] Medical Oncology Department, Centro Hospitalar Universitário de São João, 4200-319 Porto, Portugal; claramlborges@gmail.com (C.B.); daniela.psa@sapo.pt (D.A.)

[13] Cancer Research Institute, Seoul National University, 03080 Seoul, Korea

[14] Department of Surgery, Seoul National University College of Medicine, 03080 Seoul, Korea

[15] Department of Pathology, Seoul National University College of Medicine, 03080 Seoul, Korea; woohokim@snu.ac.kr (W.H.K.)

[16] Faculty of Sciences, University of Porto, 4169-007 Porto, Portugal.

* Correspondence: carlaol@i3s.up.pt; Tel.: +351-220-408-800

† Authors contributed equally.

Supplementary Methods:

Patient samples, data collection and tissue microarray preparation

Two GC patient cohorts from Portugal and South Korea, and their respective tissue microarrays (TMA) were analyzed. The Portuguese GC cohort, from Centro Hospitalar Universitário de São João (CHUSJ) in Porto, comprised samples from GC patients surgically treated between January 2008 and December 2014, and stored at the Tumor Biobank of CHUSJ/Ipatimup.[19] The South Korean GC cohort, from Seoul National University Hospital (SNUH), comprised tumor samples from GC patients, surgically treated between January 2010 and December 2011. A tissue microarray (TMA) was prepared from formalin-fixed paraffin-embedded (FFPE) tumor material from both cohorts. Representative tumor areas selected by a Pathologist were punched (2 mm diameter) and each tissue core was deposited into a recipient paraffin block using an Arraymold Kit A (IHC World, Woodstock, Maryland, USA),[20] for the Portuguese cohort, or a trephine apparatus, for the Korean cohort. Each TMA slide containing ~55 GC cores (Portuguese) and 59 GC cores (Korean), was stained with hematoxylin and eosin (H&E) for morphological confirmation of representative areas from original tumors. After excluding patients lacking clinicopathological or treatment data, patients lost to follow-up or cores without adequate tumor representation for analysis, samples from 326 patients (Portugal) and 638 patients (South Korea) were included in the analysis. From Portugal, 121/326 cases and from South Korea, 272/638 cases received platinum- and/or fluoropyrimidine-based chemotherapy in addition to surgery, mostly in an adjuvant setting (Figure S1). Clinicopathological and treatment data were retrieved from the patients' medical records in both cohorts. In Portugal, these data plus survival data were obtained at the Pathology and Surgical Departments of CHUSJ. The survival information from the South Korea cohort was request from the Ministry of Security and Public Administration according to institutional regulations. Follow-up ended in November 2016, and the median follow-up time was 29 months (Interquartile Range: 42) for the Portuguese cohort. The end of the follow-up period was September 2019 and the median follow-up time was 97 months (Interquartile Range: 52) in the South Korean cohort. These retrospective studies were approved by the Institutional Ethics Committees of CHUSJ (CES 122/15 and CES 117/18) and of SNUH (IRB:

H1706-105-860), and informed patient consent obtained from all patients or their legal representatives at both locations.

Tumor staging (pTNM - Pathological Tumor-Node-Metastasis) is reported according to the seventh edition of the American Joint Committee on Cancer (AJCC) classification system. This study was REMARK compliant.

CD44v6 immunohistochemistry of GC samples

Immunohistochemistry (IHC) staining for CD44v6 was performed in 3-μm TMA sections, using a mouse monoclonal antibody (clone MA54, 1:400 dilution for 32 min; Invitrogen, Carlsbad, California, USA). The assay was carried out on an automated Ventana BenchMark ULTRAStaining System, using the OptiView DAB IHC Detection Kit (both from Roche/Ventana Medical Systems, Tucson, Arizona, USA) according to manufacturers' instructions. Positive (human skin) and negative staining controls were performed in parallel with TMA sections. The percentage of tumor cells displaying membranous expression of CD44v6 was assessed, for each sample, by two pathologists and two researchers in a blind manner. Four categories were defined to classify the extent of CD44v6 tumor expression: "CD44v6_0" – no staining at the cell membrane; "CD44v6_1+" – membranous staining in up to 10% of tumor cells; "CD44v6_2+" – membranous staining in between 11 and 50% of tumor cells; "CD44v6_3+" – membranous staining in between 51% and 75% of tumor cells; and CD44v6_4+ – membranous staining in over 75% of tumor cells (Figure 1a).

Statistical Analysis

Patients' clinicopathological features were compared according to CD44v6 expression and treatment type. Univariate analyses for categorical variables were performed using chi-square or Fisher's exact test as appropriate. Continuous variables were analyzed using Student's t-test or one-way ANOVA with Tukey's post hoc test.

Kaplan-Meier estimates of overall survival (OS) were obtained between the groups and the Log-Rank (Mantel-Cox) test performed to identify significant differences between survival curves. Afterwards, Multivariate Cox regression analysis of OS was performed, and the hazard ratio (HR) and 95% confidence interval (CI) estimated, in order to determine factors that were

independently associated with OS. A p-value < 0.05 was considered significant. All analyses were performed using IBM SPSS Statistics versions 26 and 27 for Windows (IBM Corp, Armonk, New York, USA). This study was TRIPOD compliant.

Supplementary Figures:

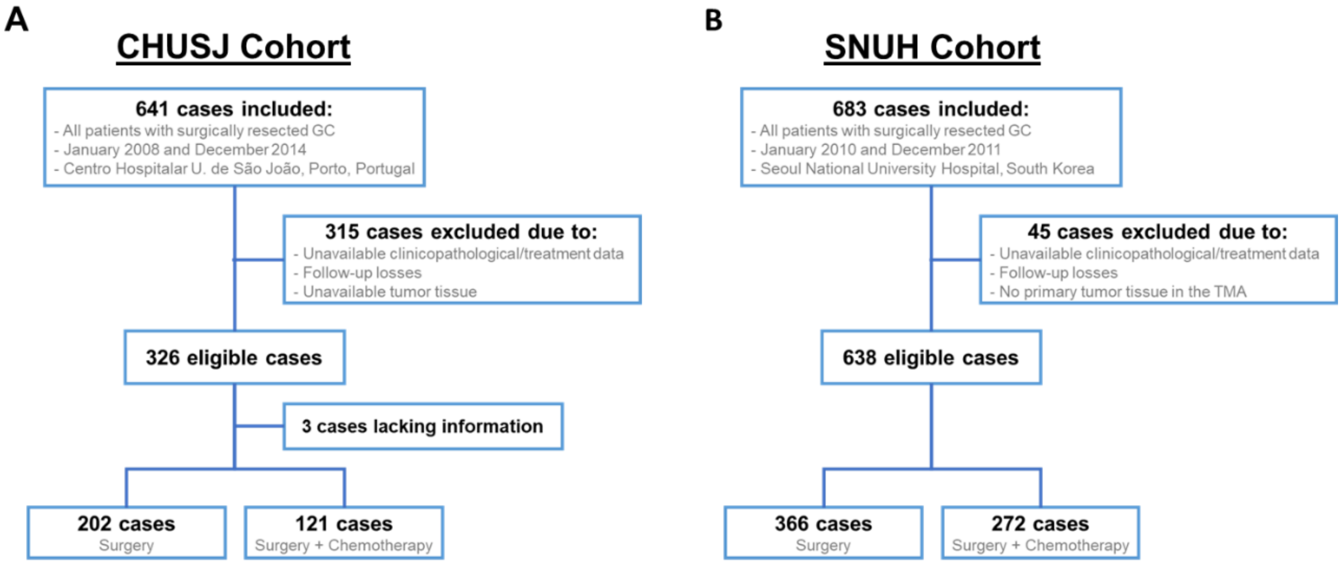

Figure S1. GC cohort profiles. **(a)** CHUSJ cohort profile. From the 326 gastric adenocarcinoma patients eligible for this study, 202 were treated with surgery and 121 patients were treated with surgery and conventional chemotherapy; **(b)** SNUH GC cohort profile. From the 638 gastric adenocarcinoma patients eligible for this study, 366 were treated with surgery and 272 were treated with both surgery and conventional chemotherapy.

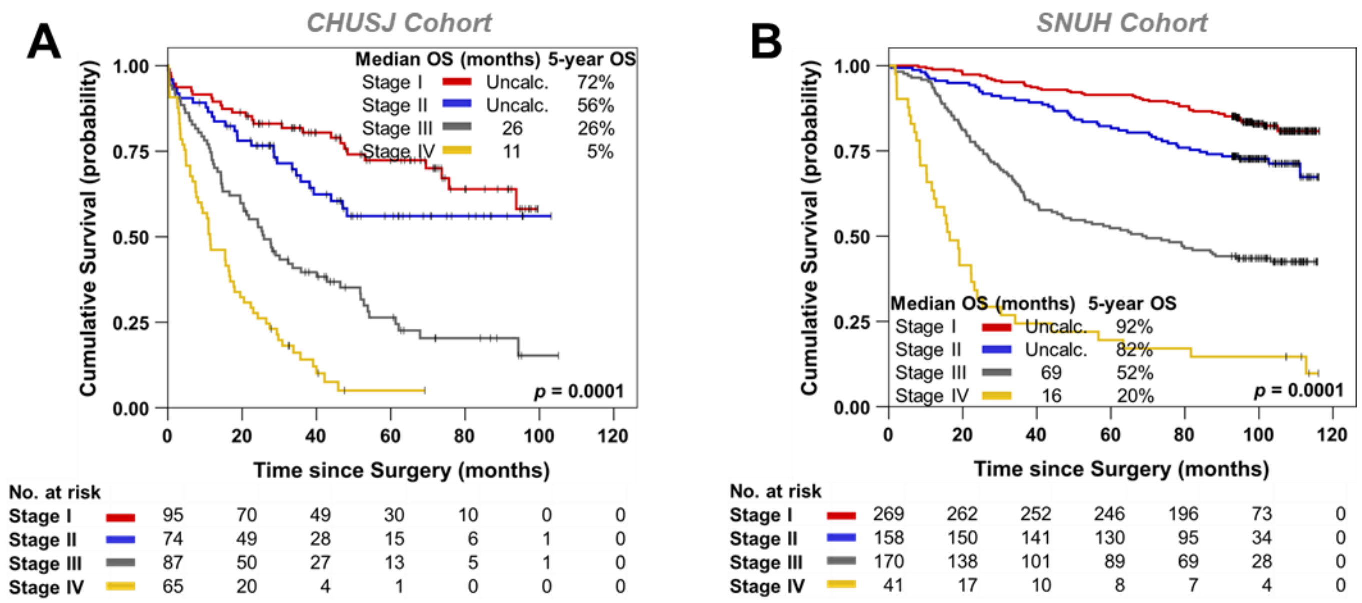

Figure S2. Kaplan–Meier estimates showing overall survival (OS) according to pTNM staging of GC patients from the two studied cohorts: **(a)** CHUSJ cohort, where, as expected, higher pTNM stages show decreased survival rates (p = 0.0001). p-values for pairwise comparisons by the Log-Rank (Mantel–Cox test) are shown in Table S2; **(b)** SNUH cohort, where, as expected, higher pTNM stages show decreased survival rates (p = 0.0001). Median OS and 5-year OS are shown for each patient subgroup. The tables below each graph indicate the number of patients still at risk in each group. p-values for pairwise comparisons by the Log-Rank (Mantel–Cox test) are shown in Table S3.

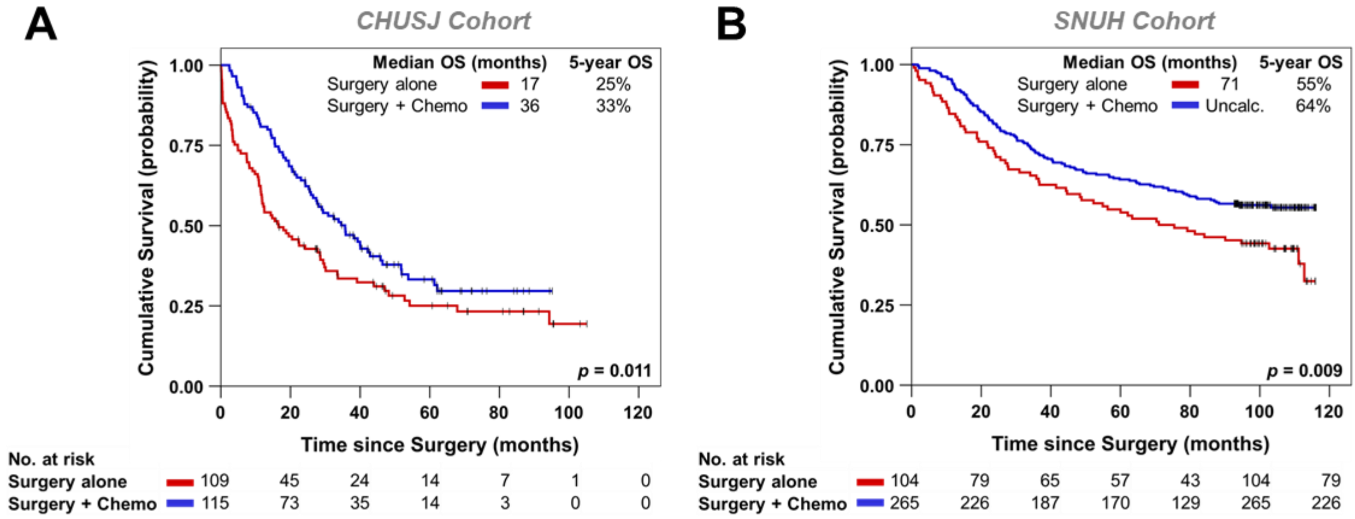

Figure S3. Kaplan–Meier estimates showing OS of GC patients from the CHUSJ cohort **(a)** and the SNUH cohort **(b)**, according to whether they were treated with surgery alone or with surgery + conventional chemotherapy. Median OS and 5-year OS are shown for each patient subgroup. The tables below each graph indicate the number of patients still at risk in each group. Since GC patients that received chemotherapy in addition to surgery were mostly from pTNM stage II to IV, when evaluating the benefit of chemotherapy on OS, pTNM stage I patients were excluded from the analysis as the majority of these patients (> 90%) had surgery alone and including them in this analysis would introduce a bias in the results.

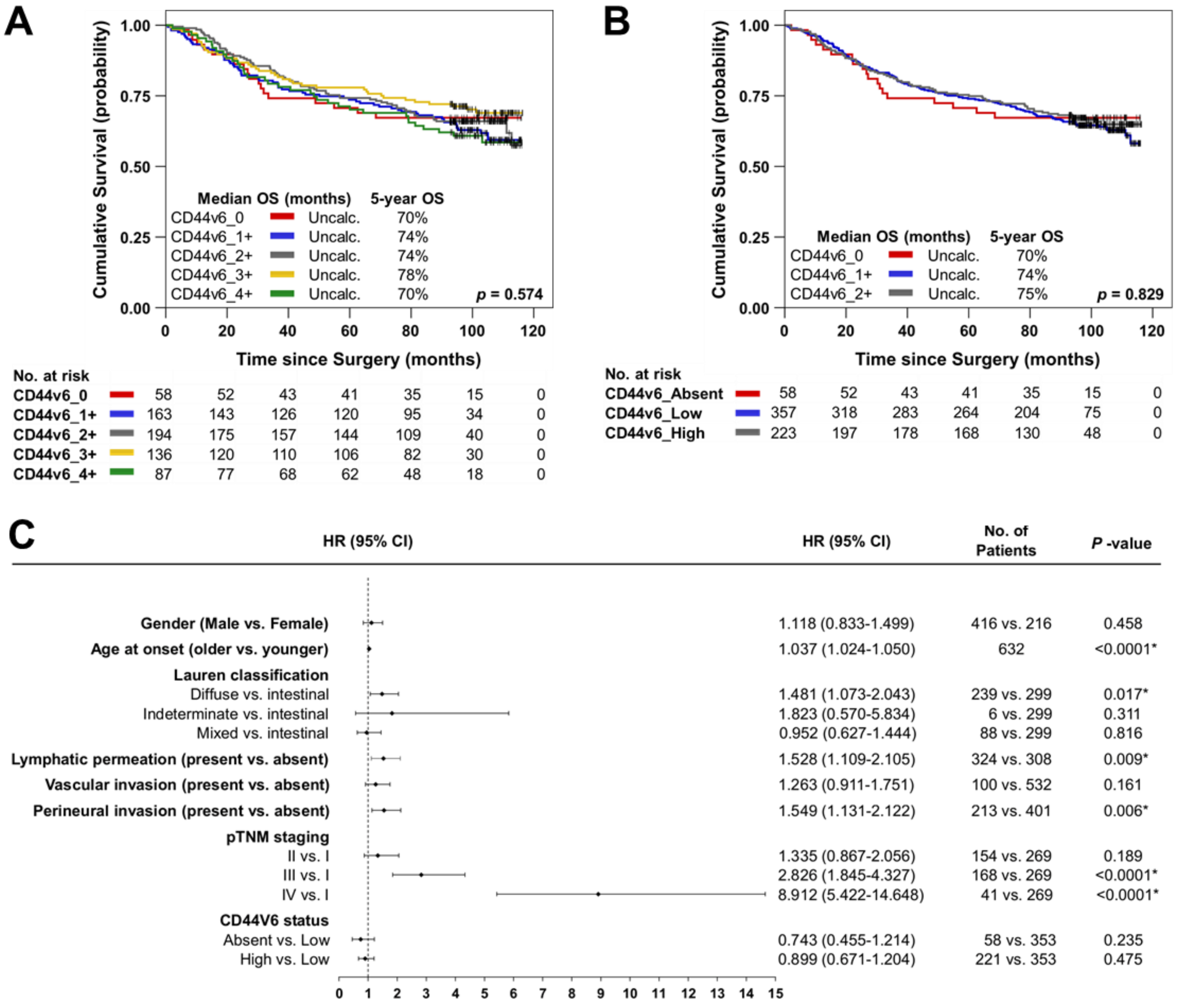

Figure S4. Kaplan–Meier estimates showing OS of GC patients, from the SNUH cohort, **(a)** according to CD44v6 sub-categories and corresponding median OS ($p = 0.574$); **(b)** Kaplan-Meier estimates showing OS of GC patients according to absent, low and high expression of CD44v6 and corresponding median OS ($p = 0.829$). Median OS and 5-year OS are shown for each patient subgroup; **(c)** Forrest plot of the multivariate analysis. * highlights statistically significant differences.

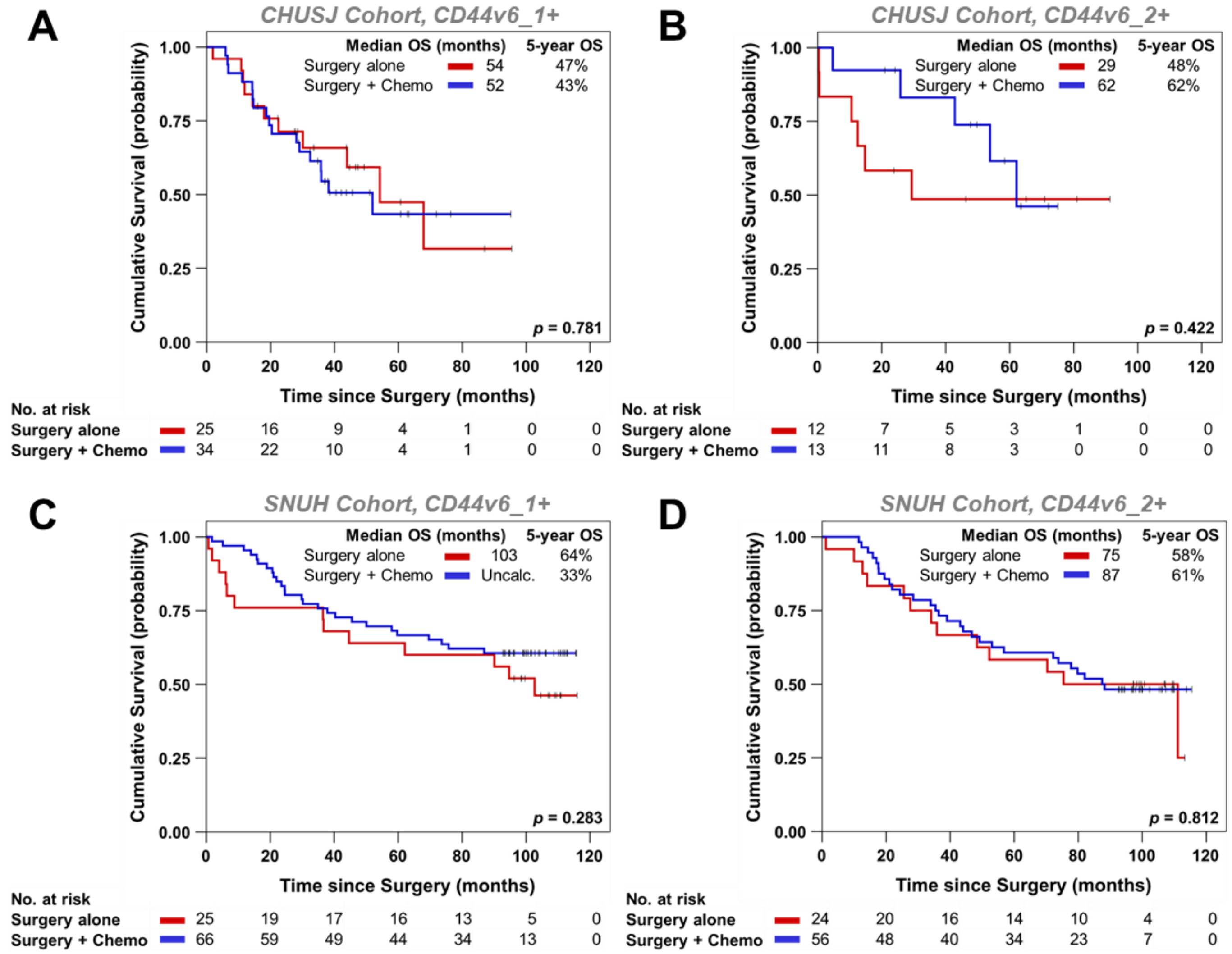

Figure S5. Kaplan–Meier estimates showing OS of GC patients, with CD44v6_1+ or CD44v6_2+ tumors, treated with surgery alone or with surgery plus conventional chemotherapy. OS of CHUSJ patients with CD44v6_1+ **(a)** or CD44v6_2+ tumors **(b)**; OS of SNUH patients with CD44v6_1+ **(c)** or CD44v6_2+ tumors **(d)**. Only pTNM stage II and III patients are included in this analysis. Median OS and 5-year OS are shown for each patient subgroup. The tables below each graph indicate the number of patients still at risk in each group.

Supplementary Tables:

Table S1. Clinicopathological characterization of the gastric cancer cohorts from CHUSJ, Portugal, and from SNUH, South Korea.

Variables	CHUSJ GC cohort (n=326)		SNUH GC cohort (n=638)	
	**No.**	**%**	**No.**	**%**
**Age (years)**				
Mean	67.7		60.8	
SD	11.8		12.3	
**Gender**				
Male	185	56.7%	420	65.8%
Female	141	43.3%	218	34.2%
M:F ratio	1.3:1		1.9:1	
**Laurén classification**			*632*1*	
Intestinal	163	50.0%	299	47.3%
Diffuse	44	13.5%	239	37.8%
Mixed	84	25.8%	88	13.9%
Indeterminate	35	10.7%	6	0.9%
**Growth pattern**				
Expansive	60	18.4%	65	10.2%
Infiltrative	252	77.3%	573	89.8%
Unclassified	14	4.3%	0	0.0%
**Wall invasion**				
Mucosa + Submucosa	74	22.7%	212	33.2%
Muscular	41	12.6%	118	18.5%
Subserosa + Serosa	199	61.0%	302	47.3%
Other organs	12	3.7%	6	0.9%
**Lymphatic permeation**	*324*1*			
Absent	99	30.6%	309	48.4%
Present	225	69.4%	329	51.6%
**Perineural invasion**	*325*1*			
Absent	166	50.9%	405	63.5%
Present	159	48.9%	233	36.5%
**Vascular invasion**	*323*1*			
Absent	131	40.6%	536	84.0%
Present	192	58.4%	102	16.0%
**Surgical margins**	*325*1*			
R0	290	89.2%	638	100.0%
R1/R2	35	10.8%	0	0.0%
**Depth of invasion (T)**				
pT1	74	22.7%	212	33.2%
pT2	41	12.6%	118	18.5%
pT3-T4	211	64.7%	308	48.3%
**Lymph node metastases**	*325*1*			
**(N)**	126	38.8%	324	50.8%
Absent (pN0)	199	61.2%	314	49.2%
Present (pN+)				
**Distant metastases (M)**				
Absent	257	78.8%	596	93.4%
Present	69	21.2%	42	6.6%
**TNM Staging**				
I	95	29.1%	269	42.2%
II	74	22.7%	158	24.8%
III	88	27.0%	170	26.6%
IV	69	21.2%	41	6.4%

*1 Remaining data not available.

Table S2. Pairwise comparisons of GC patients from the CHUSJ cohort, according to pTNM staging (from Figure S2a). The Log-Rank (Mantel-Cox) test indicates the significant differences between survival curves.

	Stage I	Stage II	Stage III	Stage IV
**Stage I**	-	0.097	< 0.0001	< 0.0001
**Stage II**	0.097	-	< 0.0001	< 0.0001
**Stage III**	< 0.0001	< 0.0001	-	< 0.0001
**Stage IV**	< 0.0001	< 0.0001	< 0.0001	-

Table S3. Pairwise comparisons of GC patients from the SNUH cohort, according to pTNM staging (from Figure S2b). The Log-Rank (Mantel-Cox) test indicates the significant differences between survival curves.

	Stage I	Stage II	Stage III	Stage IV
**Stage I**	-	0.006	< 0.0001	< 0.0001
**Stage II**	0.006	-	< 0.0001	< 0.0001
**Stage III**	< 0.0001	< 0.0001	-	< 0.0001
**Stage IV**	< 0.0001	< 0.0001	< 0.0001	-

Table S4. Pairwise comparisons of GC patients from the CHUSJ cohort, according to sub-categories of CD44v6 expression (from Figure 1b). The Log-Rank (Mantel-Cox) test indicates the significant differences between survival curves.

	CD44v6_0	CD44v6_1+	CD44v6_2+	CD44v6_3+	CD44v6_4+
**CD44v6_0**	-	0.004	0.047	0.869	0.739
**CD44v6_1+**	0.004	-	0.604	0.015	0.007
**CD44v6_2+**	0.047	0.604	-	0.105	0.060
**CD44v6_3+**	0.869	0.015	0.105	-	0.663
**CD44v6_4+**	0.739	0.007	0.060	0.663	-

Table S5. Pairwise comparisons of GC patients from the CHUSJ cohort, according to CD44v6 status (from Figure 1c). The Log-Rank (Mantel-Cox) test indicates the significant differences between survival curves.

	CD44v6_Absent	CD44v6_Low	CD44v6_High
**CD44v6_absent**	-	0.004	0.950
**CD44v6_Low**	0.004	-	0.002
**CD44v6_High**	0.950	0.002	-

Table S6. Clinicopathological associations with extent of CD44v6 expression (according to CD44v6_Absent, _Low and _High) in gastric tumors from the CHUSJ cohort.

Variables	Total No. Patients n = 326	CD44v6 Absent n = 68/326 (21%)	CD44v6 Low n = 169/326 (52%)	CD44v6 High n = 89/326 (27%)	*p*- value
**Age (years)**					
Mean	67.7	68.0	67.0	69.0	> 0.05
Standard deviation	11.8	12.3	11.8	11.5	
**Gender**					
Male	185 (56.7%)	42 (61.8%)	100 (59.2%)	43 (48.3%)	> 0.05
Female	141 (43.3%)	26 (38.2%)	69 (40.8%)	46 (51.7%)	
Male:Female ratio	1.3:1	1.6:1	1.4:1	0.9:1	
**Laurén classification**					
Intestinal	163 (50.0%)	31 (45.6%)	91 (53.8%)	41 (46.1%)	
Diffuse	44 (13.5%)	12 (17.6%)	20 (11.8%)	12 (13.5%)	> 0.05
Mixed	84 (25.8%)	18 (26.5%)	36 (21.3%)	30 (33.7%)	
Indeterminate	35 (10.7%)	7 (10.3%)	22 (13.0%)	6 (6.7%)	
**Growth pattern**					
Expansive	60 (18.4%)	13 (19.1%)	31 (18.3%)	16 (18.0%)	> 0.05
Infiltrative	252 (77.3%)	51 (75.0%)	130 (76.9%)	71 (79.8%)	
Unclassified	14 (4.3%)	4 (5.9%)	8 (4.7%)	2 (2.2%)	
**Lymphatic permeation**[*1]					
Absent	99 (30.5%)	21 (30.9%)	57 (34.1%)	21 (23.6%)	> 0.05
Present	225 (69.2%)	47 (69.1%)	110 (65.9%)	68 (76.4%)	
**Perineural invasion**[*1]					
Absent	166 (51.1%)	34 (50.0%)	97 (57.4%)	35 (39.8%)	**0.027**
Present	159 (48.9%)	34 (50.0%)	72 (42.6%)	53 (60.2%)	
**Vascular invasion**[*1]					
Absent	131 (40.6%)	25 (37.3%)	81 (48.2%)	25 (28.4%)	**0.008**
Present	192 (59.4%)	42 (62.7%)	87 (51.8%)	63 (71.6%)	
**Surgical margins**[*1]					
R0	290 (89.2%)	58 (85.3%)	159 (94.6%)	73 (82.0%)	**0.004**
R1/R2	35 (10.8%)	10 (14.7%)	9 (5.4%)	16 (9.6%)	
**Depth of invasion (T)**					
pT1	74 (22.7%)	14 (20.6%)	48 (28.4%)	12 (13.5%)	**0.015**
pT2	41 (12.6%)	6 (8.8%)	17 (10.1%)	18 (20.2%)	
pT3-T4	211 (64.7%)	48 (70.6%)	104 (61.5%)	59 (66.3%)	
**Lymph node metastases (N)**[*1]					
Absent (pN0)	126 (38.8%)	27 (39.7%)	68 (40.5%)	31 (34.8%)	> 0.05
Present (pN+)	199 (61.2%)	41 (60.3%)	100 (59.5%)	58 (65.2%)	
**Distant metastases (M)**					
Absent	257 (78.8%)	48 (70.6%)	140 (82.8%)	69 (77.5%)	> 0.05
Present	69 (21.2%)	20 (29.4%)	29 (17.2%)	20 (22.5%)	
**TNM Staging**					
I	95 (29.1%)	17 (25.0%)	55 (32.5%)	23 (25.8%)	
II	74 (22.7%)	16 (23.5%)	38 (22.5%)	20 (22.5%)	> 0.05
III	88 (27.0%)	15 (22.1%)	47 (27.8%)	26 (29.2%)	
IV	69 (21.2%)	20 (29.4%)	29 (17.2%)	20 (22.5%)	

[*1] Data not available for < than 5 cases.

Table S7. Clinicopathological associations with extent of CD44v6 expression (according to CD44v6_Absent, _Low and _High) in gastric tumors from the SNUH cohort.

Variables	Total No. Patients n = 638	CD44v6 Absent n = 58/638 (9%)	CD44v6 Low n = 357/638 (56%)	CD44v6 High n = 223/638 (35%)	*p*-value
**Age (years)**					
Mean	60.8	59.5	60.4	61.7	> 0.05
Standard deviation	12.3	12.8	12.1	12.4	
**Gender**					
Male	420 (65.8%)	39 (67.2%)	251 (70.3%)	130 (58.3%)	**0.012**
Female	218 (34.2%)	19 (32.8%)	106 (29.7%)	93 (41.7%)	
Male:Female ratio	1.9:1	2.1:1	2.4:1	1.4:1	
**Laurén classification**[*1]					
Intestinal	299 (47.3%)	26 (44.8%)	169 (47.9%)	104 (47.1%)	
Diffuse	239 (37.8%)	29 (50.0%)	126 (35.7%)	84 (38.0%)	> 0.05
Mixed	88 (13.9%)	2 (3.4%)	55 (15.6%)	31 (14.0%)	
Indeterminate[*2]	6 (0.9%)	1 (1.7%)	3 (0.8%)	2 (0.9%)	
**Growth pattern**					
Expansive	65 (10.2%)	6 (10.3%)	37 (10.4%)	22 (9.9%)	> 0.05
Infiltrative	573 (89.8%)	52 (89.7%)	52 (89.6%)	201 (90.1%)	
**Lymphatic permeation**					
Absent	309 (48.4%)	28 (48.3%)	181 (50.7%)	100 (44.8%)	> 0.05
Present	329 (51.6%)	30 (51.7%)	176 (49.3%)	123 (55.2%)	
**Perineural invasion**					
Absent	405 (63.5%)	27 (46.6%)	233 (65.3%)	145 (65.0%)	**0.019**
Present	233 (36.5%)	31 (53.4%)	124 (34.7%)	78 (35.0%)	
**Vascular invasion**					
Absent	536 (84.0%)	48 (82.8%)	301 (84.3%)	187 (83.9%)	> 0.05
Present	102 (16.0%)	10 (17.2%)	56 (15.7%)	36 (16.1%)	
**Surgical margins**[*3]					
R0	638 (100%)	58 (100%)	357 (100%)	223 (100%)	
R1/R2	0 (0.0%)	0 (0.0%)	0 (0.0%)	0 (0.0%)	
**Depth of invasion (T)**					
pT1	212 (33.2%)	11 (19.0%)	136 (38.1%)	65 (29.1%)	**0.003**
pT2	118 (18.5%)	7 (12.1%)	62 (17.4%)	49 (22.0%)	
pT3-T4	308 (48.3%)	40 (69.0%)	159 (44.5%)	109 (48.9%)	
**Lymph node metastases (N)**					
Absent (pN0)	324 (50.8%)	25 (43.1%)	192 (53.8%)	107 (48.0%)	> 0.05
Present (pN+)	314 (49.2%)	33 (56.9%)	165 (46.2%)	116 (52.0%)	
**Distant metastases (M)**					
Absent	596 (93.4%)	52 (89.7%)	339 (95.0%)	205 (91.9%)	> 0.05
Present	42 (6.6%)	6 (10.3%)	18 (5.0%)	18 (8.1%)	
**TNM Staging**					
I	269(42.2%)	15 (25.9%)	168 (47.1%)	86 (38.6%)	
II	158 (24.8%)	16 (27.6%)	81 (22.7%)	61 (27.4%)	**0.043**
III	170 (26.6%)	21 (36.2%)	90 (25.2%)	59 (26.5%)	
IV	41 (6.4%)	6 (10.3%)	18 (5.0%)	17 (7.6%)	

*1 Data not available for < than 7 cases.

Table S8. Pairwise comparisons of GC patients from the CHUSJ cohort treated with surgery alone, according to CD44v6 status (from Figure 2a). The Log-Rank (Mantel-Cox) test indicates the significant differences between survival curves.

	CD44v6_Absent	CD44v6_Low	CD44v6_High
**CD44v6_absent**	-	0.027	0.235
**CD44v6_Low**	0.027	-	0.0002
**CD44v6_High**	0.235	0.0002	-

Table S9. Pairwise comparisons of GC patients from the CHUSJ cohort treated with surgery and chemotherapy, according to CD44v6 status (from Figure 2b). The Log-Rank (Mantel-Cox) test indicates the significant differences between survival curves.

	CD44v6_Absent	CD44v6_Low	CD44v6_High
**CD44v6_absent**	-	0.025	0.126
**CD44v6_Low**	0.025	-	0.640
**CD44v6_High**	0.126	0.640	-
